# The Transcriptional Regulators of the CRP Family Regulate Different Essential Bacterial Functions and Can Be Inherited Vertically and Horizontally

**DOI:** 10.3389/fmicb.2017.00959

**Published:** 2017-05-31

**Authors:** Gloria Soberón-Chávez, Luis D. Alcaraz, Estefanía Morales, Gabriel Y. Ponce-Soto, Luis Servín-González

**Affiliations:** ^1^Departamento de Biología Molecular y Biotecnología, Instituto de Investigaciones Biomédicas, Universidad Nacional Autónoma de México, Ciudad UniversitariaMexico City, Mexico; ^2^Laboratorio de Ciencias de la Sostenibilidad, Instituto de Ecología, Universidad Nacional Autónoma de México, Ciudad UniversitariaMexico City, Mexico; ^3^Departamento de Ecología Evolutiva, Instituto de Ecología, Universidad Nacional Autónoma de México, Ciudad UniversitariaMexico City, Mexico

**Keywords:** CRP-orthologs, core-genome, pan-genome, horizontal gene transfer, bacterial-evolution

## Abstract

One of the best-studied transcriptional regulatory proteins in bacteria is the *Escherichia coli* catabolite repressor protein (CRP) that when complexed with 3′-5′-cyclic AMP (cAMP) changes its conformation and interacts with specific DNA-sequences. CRP DNA-binding can result in positive or negative regulation of gene expression depending on the position of its interaction with respect to RNA polymerase binding site. The aim of this work is to review the biological role and phylogenetic relations that some members of the CRP family of transcriptional regulators (also known as cAMP receptor protein family) have in different bacterial species. This work is not intended to give an exhaustive revision of bacterial CRP-orthologs, but to provide examples of the role that these proteins play in the expression of genes that are fundamental for the life style of some bacterial species. We highlight the conservation of their structural characteristics and of their binding to conserved-DNA sequences, in contrast to their very diverse repertoire of gene activation. CRP activates a wide variety of fundamental genes for the biological characteristic of each bacterial species, which in several instances form part of their core-genome (defined as the gene sequences present in all members of a bacterial species). We present evidence that support the fact that some of the transcriptional regulators that belong to the CRP family in different bacterial species, and some of the genes that are regulated by them, can be inherited by horizontal gene transfer. These data are discussed in the framework of bacterial evolution models.

## The Paradigm of Catabolite-Repression Regulation in *Escherichia coli*

The *Escherichia coli* catabolite repressor protein (CRP) is one of the best-studied transcriptional regulators in bacteria (Kolb et al., 1993; Gosset et al., 2004). The crystal structure of the 45 KDa CRP-protein has been determined to a 2.1 Å resolution (Passner et al., 2000; Berrera et al., 2007); it consists of a dimer that suffers an allosteric transition when binding cAMP (Popovych et al., 2009). The CRP/cAMP dimer is able to bind the 22 bp conserved DNA sequence: 5′-AAATGTGAN_6_TCACATTT-3′, which is called the CRP-binding sequence (CBS). Positions 4–8 and 15–19 in CBS (underlined) are the most important for CRP/cAMP-DNA interaction (Parkinson et al., 2011).

Based on the position of the CBS and the mechanism of transcription activation, CRP-dependent promoters can be classified into three classes. In class I promoters, the CBS is located upstream of the promoter (*lac* promoter for example), and transcriptional activation involves the interaction between CRP and the carboxy-terminal domain of RNA-polymerase (RNA-P) α-subunit (α-CTD), facilitating the binding of RNA-P to the promoter to yield the closed-complex of transcription. In class II promoters, the CBS overlaps the -35 site of the promoter (*galP1* promoter for example). At this type of promoters, CRP also recruits the RNA-P through the interaction with the α-CTD to form the closed complex and facilitates isomerization of the RNA-P into the open complex (Lawson et al., 2004). In class III promoters CRP interacts with DNA at multiple sites in combination with additional proteins (*acs*P2 promoter for example) (Beatty et al., 2003).

CRP coupled with cAMP is the main regulator of catabolite repression in Enterobacteria. This phenomenon happens when bacteria are exposed to glucose and another non-preferred sugar: in this condition the genes encoding for the enzymes responsible for the degradation of the non-preferred sugar are repressed. The activity of CRP is modulated by the availability of cAMP synthetized by the adenylate cyclase enzyme by a mechanism that is coupled with glucose transport by the phosphoenol pyruvate sugar-phosphotransfer system (PTS) (Deutscher, 2008).

Thus CRP protein represents a paradigm of a bacterial transcriptional regulator of a central metabolic pathway, since carbon catabolism in Enterobacteria is a fundamental characteristic of their biology. The central role of CRP in *E. coli* metabolism and adaptation to carbon source availability can also be appreciated when considering the dramatic change that CRP mutations cause in the global expression pattern of *E. coli* (Zheng et al., 2004; Cooper et al., 2008).

The aim of this work is to review the role that CRP-orthologs play in different bacterial species. We use the analysis of orthologs of this transcriptional regulator as a model to challenge the currently accepted theoretical frame for the evolutionary dynamics of bacterial genomes, which states that the bacterial genetic information encoded in the core genome (DNA sequences that are present in all members of a bacterial species) encode the essential function for the biology of a bacterial species, are inherited vertically and thus, represents the evolutionary history of any bacterial species.

## Phylogeny of CRP-Orthologs in Different Bacterial Species: The Role of Horizontal Gene Transfer (HGT)

The so-called pan-genome represents the genetic repertoire that a determined bacterial species possess. It consists of three parts: (i) the core genome formed by genes that are conserved among all members of a bacterial species; (ii) the accessory genome present only in a fraction of the members; and (iii) strain-specific genes, which are present only in a single genome. The accessory genome and the strain-specific genes are inherited by horizontal gene transfer (HGT) (Medini et al., 2005; Tettelin et al., 2008; Guimarães et al., 2015).

The core genome concept is defined as an intra-species concept, but since it defines the basic functions of the biology of a determined bacterial species, it is implied that it should have, as a coherent ensemble, a relation of ancestry with the core-genome of other bacterial species. This is the rationale for constructing phylogenetic trees with ribosomal RNA and other universal genes to obtain a bacterial taxonomy (Ciccarelli et al., 2006; Hug et al., 2016).

In this work we use CRP as a regulatory protein representative of the *E. coli* core genome (it is indeed present in all members of this bacterial species), that regulates important gene functions for its biology, that are also part of the core-genome, to trace its role in other bacterial species. This analysis will focus in CRP phylogeny, and in the genes that are regulated by CRP-orthologs and whether they belong to the core or the accessory genomes.

**FIGURE 1:**
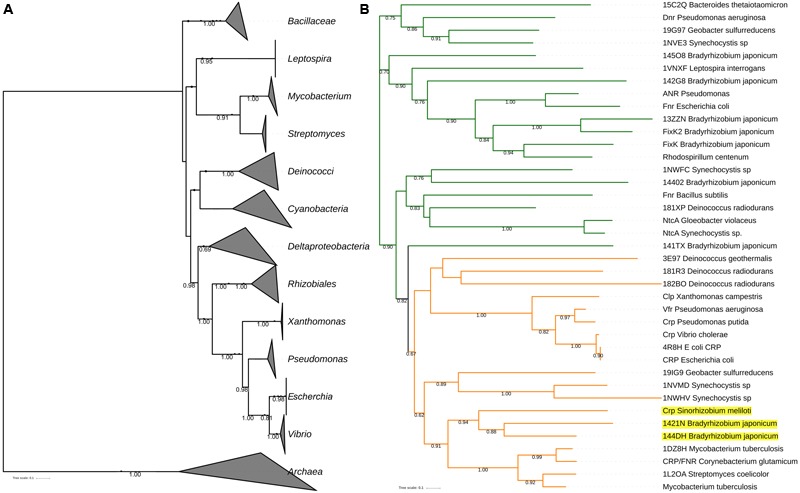
Finding horizontal gene transfer (HGT) among CRP-orthologs in different bacteria. **(A)** The Ciccarelli et al. (2006) tree of life is used as a framework to infer possible HGT-events. **(B)** The CRP/FNR phylogenetic reconstruction (details of how the tree was constructed are described in Supplementary Information); in orange are the CRP clade, where HGT events are highlighted in yellow (CRP-orthologs of *Bradyrhizobium* and *Sinorhizobium* that are grouped with Actinobacteria). Bootstrap values are shown in nodes. The FNR clade is marked in green.

The CRP-orthologs belong to the CRP/FNR super family of proteins that have a characteristic helix-turn-helix DNA-binding motif that is located in their carboxy-terminal domain (Körner et al., 2003). To determine the phylogeny of CRP-orthologs among different bacterial species, we made the reconstruction of the CRP/FNR phylogenetic tree (the description of the genes included in the phylogeny is shown in the Supplementary Information) and use as a phylogenomic reference the universal tree reported previously (Ciccarelli et al., 2006) (**Figure 1**). The great congruency of the CRP-phylogenetic tree (shown in orange in **Figure 1B**) compared with the universal tree (**Figure 1A**) shows that the gene coding for CRP-orthologs is commonly inherited by vertical transfer and thus are part of the core-genome of most bacterial groups. However, there are some incongruences that suggest that HGT has also occurred, as is the case of CRP homologous sequences of *Bradyrhizobium* and *Sinorhizobium* that are grouped with Actinobacteria (**Figure 1B**).

In the case of the CRP-orthologs that were inherited by HGT it can be concluded that the gene encoding for this transcriptional regulator is part of its accessory-genome, even though this transcriptional regulator is involved in the expression of fundamental bacterial functions as will be described below. The existence of genes encoding for fundamental functions that are inherited by HGT, and thus forming part of the accessory-genome, has already been reported in the case of *Azotobacter vinelandii* (González-Casanova et al., 2014).

## Structural Characteristics of Different CRP-Orthologs

The CRP-orthologs share important structural characteristics, as can be seen by the examples listed in **Table 1**. Furthermore, it is apparent that even CRP’s that belong to distantly related bacteria such as *E. coli, Corynebacterium glutamicum* and *Mycobacterium tuberculosis* have a highly conserved active site with the same amino-acids participating^1^ (Supplementary Figure S1).

**Table 1:** Characteristics of CRP-orthologs in different bacterial species.

Bacteria	Phylogenetic group	Protein	Consensus DNA binding site similar to: TGTGA-N_6-_TCACA	*E. coli* complementation	% identity with *E. coli* CRP (aa)	Physiological role	Reference
*Escherichia coli*	γ-proteobacteria	CRP	Yes	Yes	100	Catabolite repression	Gosset et al., 2004; Zheng et al., 2004
*Vibrio cholerae*	γ-proteobacteria	CRP	Yes	Yes	95	Virulence factors production, quorum-sensing. competence, biofilm formation, chitin utilization	Liang et al., 2007; Fong and Yildiz, 2008; Blokesch, 2012
*Pseudomonas aeruginosa*	γ-proteobacteria	Vfr^1^	Yes	Yes	61	Virulence factors production, quorum-sensing	Albus et al., 1997; Beatson et al., 2002; Fuchs et al., 2010
*Pseudomonas putida*	γ-proteobacteria	CRP	Yes	Yes	62	Assimilation of dipeptides; aromatic amino acid degradation	Milanesio et al., 2011; Herrera et al., 2012
*Xanthomonas campestris*	γ-proteobacteria	Clp^2^	Yes	Yes	45	Regulation of pathogenicity, quorum-sensing. Xanthan and biofilm production.	Chin et al., 2010; Chou, 2011; Lu et al., 2012
*Sinorhizobium meliloti*	α-proteobacteria	Clr^4^	Yes	NR	23^5^	Osmotic stress response, swimming motility	Tian et al., 2012; Krol et al., 2016
*Rhodospirillum centenum*	α-proteobacteria	CgrA^4^	Yes	NR	22	Cyst development	Marden et al., 2011; Roychowdhury et al., 2015
*Mycobacterium tuberculosis*	Actino bacteria	CRP^Mt^	Yes	Yes	32	Pathogenesis transition between replicating and non-replicating states	Akhter et al., 2007, 2008
*Streptomyces coelicolor*	Actino bacteria	CRP^Sco^	No	No	28	Secondary metabolism, antibiotic production, development control	Derouaux et al., 2004; Gao et al., 2012
*Corynebacterium glutamicum*	Actino bacteria	GlxR	Yes	Yes	29	Glyoxylate bypass, amino-acid synthesis regulation	Kim et al., 2004; Tauch and Kohl, 2009

*^1^ Binds DNA without cAMP in some promoters.*

*^2^It does not bind cAMP, but cyclic di-GMP.*

*^3^Binds both cAMP and c GMP.*

*^4^Binds cGMP.*

*^5^Blast vs. *Sinorhizobium*/Ensifer group.*

*NR, not reported.*

It is surprising that the ability to bind sequences similar to *E. coli* CBS is conserved in γ-proteobacteria, *S. meliloti* and Actinobacteria that are phylogenetically very distant (**Figure 1** and **Table 1**). CRP^Sco^ from *Streptomyces coelicolor* does not have the ability to bind *E. coli* CBS, since it presents a different mechanism of DNA-binding (Derouaux et al., 2004). Furthermore, the genes coding for all these CRP-orthologs, with the exception of CRP^Sco^ of *S. coelicolor*, and possibly *S. meliloti* Clr that have not been reported, are able to complement an *E. coli crp* mutant for carbon assimilation (**Table 1**).

It is striking that *M. tuberculosis* CRP^Mt^ and *C. glutamicum* GlxR, that only share 32 and 28% amino acid identity with *E. coli* CRP (**Table 1**), are able to interact with cAMP, producing the required allosteric changes to interact with *E. coli* CBS and RNA-P to activate all the promoters involved in catabolite repression (Franchini et al., 2015). These CRP-orthologs have conserved active sites^1^ (Supplementary Figure S1).

The CRP-orthologs from different bacterial species have different cAMP binding-affinities, which correlate with subtle changes in their structure (Stapleton et al., 2010; Serate et al., 2011; Arce-Rodríguez et al., 2012). These differences in cAMP-binding affinity have been associated with the concentration of cAMP that is produced by each bacterial species and the genes that are regulated by these transcriptional factors (Green et al., 2014).

The structural characterization of Clp from *Xanthomonas campestris* showed that it presents a conformation that enable DNA-binding to its target sequence in the absence of any nucleotide, and when it interacts with cyclic-di-GMP it dissociates from DNA (Chou, 2011).

The *Pseudomonas aeruginosa* CRP-ortholog, called Vfr, (virulence factor regulator) is able to bind to certain CBS variants and to activate transcription in the absence of cAMP (this is the case of the *lasR* promoter), but in most instances it requires the interaction with cAMP to activate transcription (Kanack et al., 2006; Fuchs et al., 2010). The Vfr cAMP-dependent transcriptional-regulation activity is inhibited by cyclic-di-GMP (Almblad et al., 2015). The Vfr crystal structure has been determined and is very similar to that of *E. coli* CRP (Cordes et al., 2011), but it has important functional differences with respect to ligand sensing and ability to interact with DNA (Serate et al., 2011).

The structural and functional characterization of CRP-orthologs shows that they possess highly conserved structures that in different bacterial species maintain their ability to recognize sequences similar to the *E. coli* CBS and that their interaction with nucleotides, mainly cAMP, constitutes a way of regulating their activity by conformational changes.

## Biological Role of CRP Orthologs in Different Bacterial Species

The repertoire of biological functions that CRP-orthologs modulate through transcriptional regulation is very wide (**Table 1**). In all cases the genes that are regulated by CRP-orthologs, encode functions that are very important for the biology of each bacterial species, even though they do not form part of their primary metabolism (**Table 1**).

We will give a brief description of the biological functions that are regulated by CRP-orthologs in the bacterial species shown in **Table 1**, highlighting some comparison among closely related species.

The CRP protein of *Vibrio cholerae* (Skorupski and Taylor, 1997) has 95% amino-acid identity with *E. coli* CRP, and besides regulating catabolite repression, it activates the transcription of genes involved in chitin degradation and competence (Blokesch, 2012), as well as genes involved in biofilm formation (Fong and Yildiz, 2008) and intestinal colonization as those involved in motility and quorum-sensing (QS), the complex regulatory cascade that regulates the expression of virulence associated traits (Liang et al., 2007). Chitin degradation is an important ecological trait that enables bacteria of the genus *Vibrio* to colonize marine organisms, while virulence associted traits are fundamental biological characteristics of *V. cholerae*. Thus, *V. cholerae* CRP presents the ability to activate the expression of different genes compared to the repertoire of genes expressed by the *E. coli* CRP; hence some of the genes expressed by *V. cholerae* CRP do not belong to the Enterobacteria core-genome.

Several *P. aeruginosa* virulence associated traits are coordinately expressed by a complex QS response. Two of the main QS transcriptional regulators, LasR and RhlR, are regulated at the level of transcription by Vfr (Albus et al., 1997; Croda-García et al., 2011). In addition, Vfr regulates *P. aeruginosa* twitching motility (Beatson et al., 2002), and the type III secretion system (Marsden et al., 2016). Vfr not only modulates the virulence of this bacterium (Wolfgang et al., 2003), but it also has a global efect on gene expression (Suh et al., 2002).

*P. aeruginosa* genes encoding for both the QS-regulators and the genes encoding virulence-associated traits, can be considered as forming part of its core genome, since they are present in all isolates belonging to this species, but they represent another example of genes that encode for fundamental biological traits that were acquired by HGT, since they are not present in other members of the genus *Pseudomonas*, such as the closely related species *P. putida* (**Table 1**). *P. aeruginosa* virulence associated traits do not form part of genomic islands and are interdisperse in the chromosome.

*P. putida* CRP regulates the expression of genes involved in the degradation of aromatic amino acids (Herrera et al., 2012) and in the utilization of some dipeptides, but the mutation of the gene coding for this transcriptional regulator has a reduced effect on the bacterial phenotype (Milanesio et al., 2011).

The comparison of *P. aeruginosa* Vfr with *P. putida* CRP reflects the case of two homologous proteins from two closely related bacteria (**Figure 1** and **Table 1**) that show very different effects in the regulation of gene expression.

*Xanthomonas campestris* is a plant pathogenic bacterium that produces the exopolysaccharide (EPS) xanthan. The *X. campestris* CRP-ortholog Clp as discussed above has peculiar structural characteristics (Chou, 2011). Clp regulates the expression of genes involved in xanthan production (Chen et al., 2010), in virulence (Chin et al., 2010), and in biofilm formation (Lu et al., 2012).

Clr is the *S. meliloti* CRP-ortholog that can be activated both by cAMP as well as by cGMP (Krol et al., 2016); it has been shown to directly activate genes involved in EPS synthesis and osmotic stress-response (Krol et al., 2016), and to participate in epidermal infection in the *S. meliloti*–*Medicago* symbiosis (Tian et al., 2012). The *clr* gene was inherited by HGT (**Figure 1**)

There are three documented examples of the function of CRP-orthologs in Actinobacteria (**Figure 1** and **Table 1**). CRP^Sco^, is present in *S. coelicolor* and is involved in the expression of genes involved in antibiotic production and in the control of differentiation (Derouaux et al., 2004; Gao et al., 2012), while *C. glutamicum* GlxR directly regulates the genes involved in the glyoxylate bypass (Kim et al., 2004; Kohl et al., 2008) and participate in the regulation of the synthesis of amino acids (Tauch and Kohl, 2009). In the case of *M. tuberculosis*, CRP^Mt^ plays a role in the regulation of the expression of genes involved in pathogenicity (Akhter et al., 2007, 2008) and will be discussed below in more detail.

The example of the three Actinobacterial CRP-orthologs shows that they constitute global regulators of genes that are important for the biology of each of these bacterial species.

To determine whether the genes regulated by *M. tuberculosis* CRP^Mt^, that are involved in pathogenicity were encoded in genomic islands, or whether they belong to the core-genome we looked for the presence of genomic islands in the chromosome of strain H37Rv, using two bioinformatics tools: IslandViewer 3 (Dhillon et al., 2015) and Zisland Explorer (Wei et al., 2016). The former program identified two islands, while the latter did not identify any. The size of the identified genomic island was of 11,482 base pairs that encode 10 genes (Supplementary Table S1). The sequences of the 60 genes that are regulated by CRP^Mt^ (Akhter et al., 2008) were BLAST-searched (Altschul et al., 1990) in the genomic islands, but none of them was found in these locations. These results show that the genes regulated by CRP^Mt^ encode traits that are not present in genomic islands, and thus seem to be important for *M. tuberculosis* biology.

## The Case of the CRP-Family of Transcriptional Regulators in the Framework of Different Bacterial Evolution Models

The role of transcriptional regulators in bacterial evolution has been discussed in the literature (Madan Babu et al., 2006; Perez and Groisman, 2009; Wang et al., 2011), and there are some conclusions that coincide with the analysis presented in this work of CRP-orthologs in some bacterial species. For example, it has been reported that transcriptional factors evolve independently of their target genes (Madan Babu et al., 2006), that species-specific genes are controlled by ancestral transcriptional factors (Perez and Groisman, 2009), and the role of HGT and the participation of mobile genetic elements has been highlighted in the formation of new regulatory networks during bacterial speciation (Wang et al., 2011). However, these explanations do not give a theoretical framework for bacterial evolution that can support the observed pattern of gene variation among bacterial species.

We have shown in this work that the CRP-orthologs of closely related bacteria as *E. coli* and *V. cholerae*, or *P. aeruginosa* and *P. putida*, can regulate very different sets of genes that usually are important for the bacterial species life style, and are conserved among the members of a specific species, but are inherited by HGT. This is the case of *P. aeruginosa* QS-regulated genes that are not present in any other *Pseudomonas* species.

We have postulated (González-Casanova et al., 2014) that bacterial evolution can be explained in the light of a probabilistic model (Blath et al., 2013, 2015). This model states that organisms that produce dormant forms that persist for unbounded periods that are much larger than their generation time (strong seed bank effect) will show a very high genetic variability and will produce a genetic pool that will be protected to some degree from genetic drift and other classical evolutionary forces. Bacteria can produce non-replicating forms, such as spores and cysts, and part of their genomes remain without being expressed forming part of phages, and thus fulfill the premises of this probabilistic model. Considering that bacteria present a strong seed bank effect, and that they show a high frequency of HGT among them, the evolution of these organisms cannot be described by classical population genetics models. One of the predictions that was made using this theoretical framework is that genes that are fundamental for the biology of a bacterial species might be inherit by HGT and thus form part of the accessory genome. We analyzed the case of *A. vinelandii* and showed this to be the case (González-Casanova et al., 2014).

In the analysis that we have presented in this work we have discusses that in several cases CRP-orthologs regulate the expression of genes that are fundamental to the biology of a bacterial species, and that the CRP target-genes are inherited by HGT, and are thus part of the accessory genome. This is the case of genes that encode virulence related traits in *V. cholerae, P. aeruginosa* and *M. tuberculosis*, which are regulated by CRP-orthologs. It is important to point out that none of these bacterial species produce spores of cysts, but present the predicted phenomenon that was previously described for *A. vinelandii* (González-Casanova et al., 2014).

The critical analysis of the reported characteristics of CRP-orthologs of different bacterial, presented in this work, enables us to conclude that even in the case of this family of very well conserved transcription regulators, it is apparent that bacterial phylogenomics cannot be explained using the theoretical frameworks that sustain classic population genetics.

## Author Contributions

GS-C conceived and designed the work. LA, GP-S, and LS-G participate in the bioinformatics analysis. EM and GP-S are responsible for the acquisition of the data. GS-C, LA, LS-G, EM, and GP-S made substantial contributions for the analysis and interpretation of the data. GS-C, LS-G, EM, and GP-S participate in drafting the work and revised it critically. GS-C, LA, LS-G, EM, and GP-S approved the final version of the work and agree to be accountable for its content.

## Conflict of Interest Statement

The authors declare that the research was conducted in the absence of any commercial or financial relationships that could be construed as a potential conflict of interest. The reviewer TT and handling Editor declared their shared affiliation, and the handling Editor states that the process nevertheless met the standards of a fair and objective review.

## Footnotes

1 http://www.rcsb.org/pdb/explore/jmol.do?structureId=4R8H&bionumber=1
