# Supplementary material for: The Transcriptional Regulators of the CRP Family Regulate Different Essential Bacterial Functions and Can Be Inherited Vertically and Horizontally
Source: Front Microbiol. 2017 May 31;8:959. doi: 10.3389/fmicb.2017.00959 (PMC5449483; doi:10.3389/fmicb.2017.00959)
Supplement: Supplementary file 1 [file Presentation_1.PDF]

Supplementary information:

The transcriptional regulators of the CRP family regulate different essential bacterial functions and can be inherited vertically and horizontally

Gloria Soberón- Chávez[1]*, Luis David Alcaraz[2], Estefanía Morales[1], Gabriel Yaxal Ponce-Soto[3], Luis Servín-González[1]

[1] Departamento de Biología Molecular y Biotecnología, Instituto de Investigaciones Biomédicas, Universidad Nacional Autónoma de México, Ciudad Universitaria, 04510, México, D. F. México.

[2] Laboratorio de Ciencias de la Sostenibilidad, Instituto de Ecología, Universidad Nacional Autónoma de México, Ciudad Universitaria, 04510 México, DF, México.

[3] Departamento de Ecología Evolutiva, Instituto de Ecología, Universidad Nacional Autónoma de México, Ciudad Universitaria, 04510 México, DF, México.

*** Correspondence:**

Gloria Soberón-Chávez

gloria@biomedicas.unam.mx

Crp/Fnr phylogenetic reconstruction.

The data and metrics of the phylogenetic tree shown in figure 1 were obtained using the following criteria:

E. coli Crp protein (4R8H)[1] was downloaded from the Protein Data Bank[2]. Through structural comparison, we selected *Deinococcus geothermalis* (3E97)[3] to perform a structural pairwise alignment and use it as the structural seed for MAFFT amino acid alignments [4]. Then Phylome database[5] Phy0035PBZ CRP reference tree was used as guide to select representative CRP/FNR sequences:

Phy0035PBZ_ECOL, Phy0013ZM2_BRAJ, Phy001FSKG_PSEA, Phy0035POF_ECOL, Phy0013ZZN_BRAJ, Phy00140FT_BRAJ, Phy001421N_BRAJ, Phy00144DH_BRAJ, Phy001DZ8H_MYCT, Phy001L2OA_STRC, Phy0019G97_GEOS, Phy001NVE3_SYNY, Phy00181R3_DEIR, Phy00181XP_DEIR, Phy0019IG9_GEOS, Phy00145O8_BRAJ, Phy0056UFS_PSEA, Phy0019KKA_GLOV, Phy001NUT8_SYNY, Phy001NWHV_SYNY, Phy001564H_BACS, Phy001NWFC_SYNY, Phy0014402_BRAJ, Phy00141TX_BRAJ, Phy001VNXF_LEPI, Phy0015C2Q_BACT, Phy00182BO_DEIR, Phy00142G8_BRAJ.

Additionally, manually selected CRP orthologs: *E. coli* WP_001615266.1, *P. aeruginosa* WP_003085214.1, *P. putida* WP_017148058.1, *V. cholera* WP_050906271.1, *X. campestris* WP_011035725.1, *S. meliloti* WP_014526586.1, *R. centenum* WP_012568132.1, *M. tuberculosis* CNF78180.1, *S. coelicolor* WP_003975365.1, *C. glutanicum* WP_003855810.1.

We estimated the Gamma parameter which has a value of 4.257, a proportion of invariant sites of 0.008, and the substitution model were estimated using the Whelan-Goldman (WAG+G) [6] model. A Maximum Likelihood (ML) tree was computed with a maximum Log likelihood value of -18275.818 with 1000 bootstrap replicas. An ML relative test time was conducted on the dataset. The alignment involved 39 amino acid sequences, eliminating all gap and missing data positions. These phylogenetic reconstructions were done in FastTree (Price et al., 2010). We are using phylogenomic reference tree of life published by Ciccareli and collaborators (2006). Phylogenetic trees were pruned and edited using the Interactive Tree of Life web application (ITOL) (Price et al., 2010).

Figure S1. Amino acid alignment of *Escherichia coli*, *Vibrio cholera, Pseudomonas aeruginosa, Pseudomonas putida, Xhantomonas campestris, Bradirhizobium japonicum* and *Deinoccocus radiodurans* CRP-orthologs; and schematic representation of the CRP-active sites of these proteins. These diagrams were obtained in the Protein Data Base (http://www.rcsb.org/pdb/explore/jmol.do?structureId=4R8H&bionumber=1).

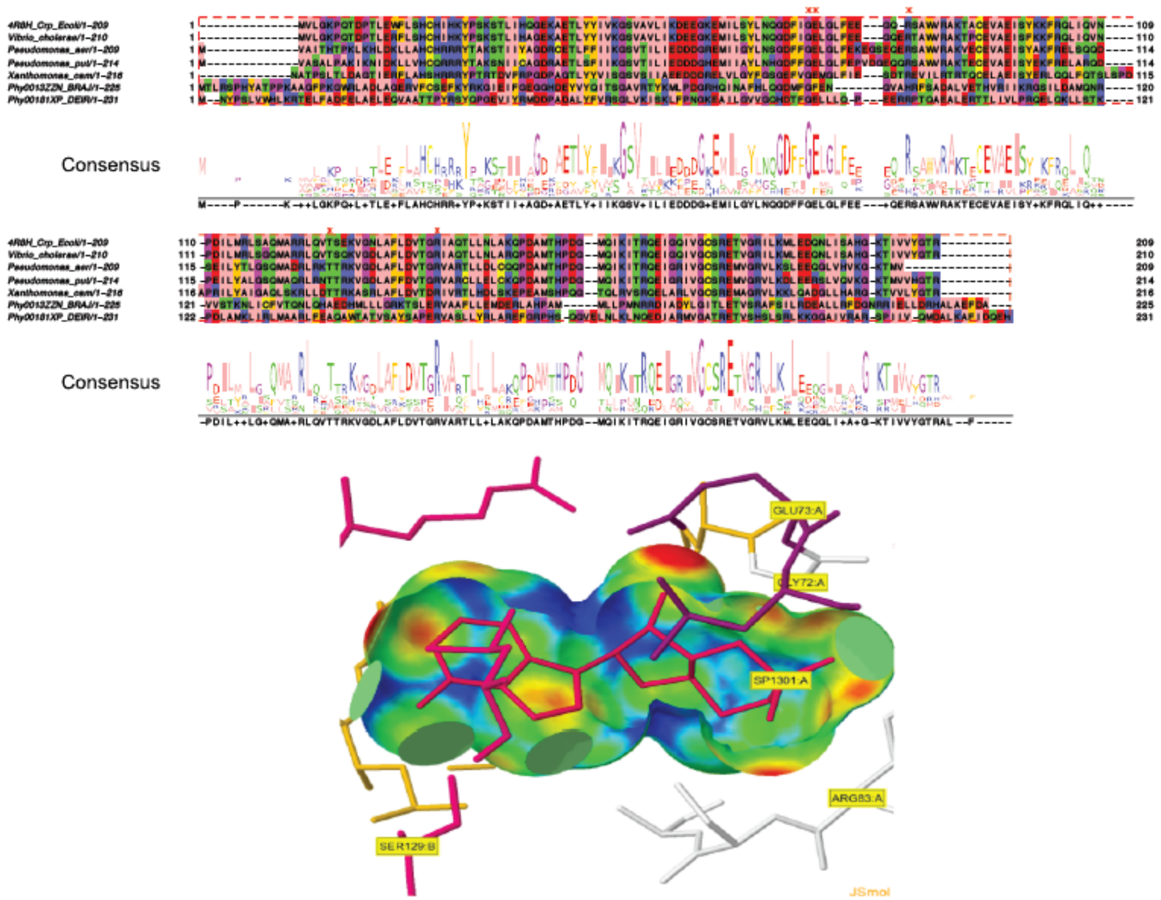

Table S1

Island start	Island end	Length	Method	Gene name	Locus	Gene start	Gene end	Strand	Product
1098845	1103318	4473	SIGI-HMM	WP_003915882.1	LH57_RS05240	1099584	1102151	1	adhesion component ABC transporter permease
1098845	1103318	4473	SIGI-HMM	WP_003898675.1	LH57_RS05245	1102158	1103318	1	hypothetical protein
1686369	1693378	7009	SIGI-HMM	WP_003898901.1	LH57_RS07995	1687409	1688230	1	hypothetical protein
1686369	1693378	7009	SIGI-HMM	WP_003911545.1	LH57_RS08000	1688443	1689342	1	hypothetical protein
1686369	1693378	7009	SIGI-HMM	WP_003918265.1	LH57_RS08005	1689515	1689955	-1	TDP-4-oxo-6-deoxy-D-glucose aminotransferase
1686369	1693378	7009	SIGI-HMM	WP_014390141.1	LH57_RS08010	1690064	1690627	-1	TDP-4-oxo-6-deoxy-D-glucose aminotransferase
1686369	1693378	7009	SIGI-HMM	WP_003407619.1	LH57_RS08015	1690800	1691465	-1	hypothetical protein
1686369	1693378	7009	SIGI-HMM	WP_003898904.1	LH57_RS08020	1691462	1691980	-1	SAM-dependent methyltransferase
1686369	1693378	7009	SIGI-HMM	WP_003407623.1	LH57_RS08025	1692246	1692941	-1	hypothetical protein
1686369	1693378	7009	SIGI-HMM	WP_003900353.1	LH57_RS08030	1692875	1693378	1	hypothetical protein

References

Berman H. M. (2000) The Protein Data Bank. Nucleic Acids Res. 28: 235–242.

Ciccarelli F D, Doerks T, von Mering C, Creevey C J, Snel B, Bork P (2006) Toward automatic reconstruction of a highly resolved tree of life. Science 311(5765)**:** 1283–1287.

Goldman N, Whelan S (2000) Statistical tests of gamma-distributed rate heterogeneity in models of sequence evolution in phylogenetics. Mol Biol Evol 17: 975–978.

Huerta-Cepas J, Bueno A, Dopazo J, Gabaldón, T (2008). PhylomeDB: a database for genome-wide collections of gene phylogenies. Nucleic Acids Res. 36: D491-496.

Katoh K, Standley D M (2013) MAFFT Multiple Sequence Alignment Software Version 7: Improvements in Performance and Usability. Mol. Biol. Evol. 30: 772–780.

Letunic I, Bork P.(2011) Interactive Tree Of Life v2: online annotation and display of phylogenetic trees made easy. Nucleic Acids Res. 39: W475-478.

Price M N, Dehal P S, Arkin, A.P. 2010. FastTree 2 – Approximately Maximum-Likelihood trees for large alignments. PLoS ONE 5(3): e9490. doi:10.1371/journal.pone.0009490.

Tamura K, Stecher G, Peterson D, Filipski A, Kumar S (2013) MEGA6: Molecular Evolutionary Genetics Analysis version 6.0. Mol. Biol. Evol. 30: 2725–2729.

Townsend P D, Rodgers T L, Glover L C, Korhonen H J, Richards S A, Colwell L J, *et al.* (2015) The Role of Protein-Ligand Contacts in Allosteric Regulation of the *Escherichia coli* Catabolite Activator Protein. J. Biol. Chem. 290: 22225–22235.
